# Building an Early Warning System for Depression: Rationale, Objectives, and Methods of the WARN-D Study

**DOI:** 10.32872/cpe.10075

**Published:** 2023-09-29

**Authors:** Eiko I. Fried, Ricarda K. K. Proppert, Carlotta L. Rieble

**Affiliations:** 1 Department of Clinical Psychology, Leiden University, Leiden, The Netherlands; Philipps-University of Marburg, Marburg, Germany

**Keywords:** ecological momentary assessment, digital health, student mental health, depression, prediction, early warning system, prevention

## Abstract

**Background:**

Depression is common, debilitating, often chronic, and affects young people disproportionately. Given that only 50% of patients improve under initial treatment, experts agree that prevention is the most effective way to change depression’s global disease burden. The biggest barrier to successful prevention is to identify individuals at risk for depression in the near future. To close this gap, this protocol paper introduces the WARN-D study, our effort to build a personalized early warning system for depression.

**Method:**

To develop the system, we follow around 2,000 students over 2 years. Stage 1 comprises an extensive baseline assessment in which we collect a broad set of predictors for depression. Stage 2 lasts 3 months and zooms into participants’ daily experiences that may predict depression; we use smartwatches to collect digital phenotype data such as sleep and activity, and we use a smartphone app to query participants about their experiences 4 times a day and once every Sunday. In Stage 3, we follow participants for 21 months, assessing transdiagnostic outcomes (including stress, functional impairment, anxiety, and depression) as well as additional predictors for future depression every 3 months. Collected data will be utilized to build a personalized prediction model for depression onset.

**Discussion:**

Overall, WARN-D will function similarly to a weather forecast, with the core difference that one can only seek shelter from a thunderstorm and clean up afterwards, while depression may be successfully prevented before it occurs.

## Highlights

- Prevention of depression in students may help prevent a lifetime of chronic illness.
- But we do not currently know whom to target in prevention programs, and when exactly.
- We introduce the protocol of the WARN-D study, aimed at building an early warning system for depression in students.
- To do so, we leverage advances in theory (complexity science), measurement (smartphone, smartwatch, and registry data), and statistical modelling (machine learning, network models).

Depressive disorders are prevalent, debilitating, and costly, and therefore among the most pressing health problems of modern living. They affect around 300 million people worldwide (Arias-de la Torre et al., 2021; Ferrari et al., 2013; James et al., 2018), are the leading cause of disability in the world, and are among the leading causes of global disease burden (Lopez et al., 2006; Mathers & Loncar, 2006). Major Depressive Disorder (MDD) is the strongest predictor for suicide (Berman, 2009), with 1 million lives lost annually (World Health Organization, 2019). Being depressed worsens the impact of common diseases like cancer and cardiovascular disease (Cuijpers et al., 2012), and about 60% of people living with depression report severe, long-lasting impairment of functioning, compromising the capacity for self-care and independent living (Kessler, Chiu, et al., 2005; Mathers & Loncar, 2006; Murray & Lopez, 1996). MDD is often chronic: Over half of depressed patients will develop multiple episodes, and many will spend a considerable part of their lifetime in a state of emotional agony and despair (Cuijpers et al., 2012).

Compared to progress in treating diseases like cancer (Biemar & Foti, 2013), breakthroughs for treating depression have lagged far behind. Treatment effectiveness has remained stable over the last decades (Khan & Brown, 2015). Around half of patients remain depressed following initial treatment with psychological therapies or pharmacotherapy (Cuijpers et al., 2018; Khan & Brown, 2015), and treatments reduce only one-third of the disease burden (van Zoonen et al., 2014). Mechanisms underlying MDD remain largely opaque, despite considerable efforts and investments into trying to understand biological underpinnings (Kapur et al., 2012; Rogers, 2017).

It is for these reasons that experts agree that *prevention*—stopping depression before it occurs—is the most important way forward to make a real difference in people’s lives (Cuijpers et al., 2012; Muñoz et al., 2010). Since 60-75% of all mental health problems develop before the age of 24, young people are an especially important group for prevention (Kessler, Berglund, et al., 2005; Solmi et al., 2022). While some progress has been made in developing and testing prevention programs that can effectively lower incidence rates by levels considered clinically relevant, improving prevention crucially relies on the reliable detection of specific individuals at risk for depression in the near future, which is currently not possible (Cuijpers et al., 2012; Muñoz et al., 2010; van Zoonen et al., 2014).

The study we describe here aims to tackle one of the largest barriers to implementing successful, tailored prevention programs: knowing when to intervene, and in which people. We address this problem by developing the personalized early warning system WARN-D. In the following, we will introduce the guiding principles of WARN-D; discuss the challenges of conceptualizing and measuring depression; describe the design, procedure, and measures of WARN-D; and conclude with strengths and challenges of the study.

## Principles Guiding the Development of WARN-D

Our study’s design, methods, and measures are guided by 6 primary goals and principles.

First, our most ambitious goal is to identify at-risk individuals before they transition into depression. We hope that our efforts will result in the first personalized early warning system for depression.

Second, we will develop this system in and for students, because timely detection of depression onset in young people promises to enable prevention programs to alleviate a potential lifetime of suffering for many, given the often-chronic nature of MDD. Students are at considerable risk for developing depression and comorbid mental health problems (Auerbach et al., 2016; Ebert et al., 2019), and the recent WHO World Mental Health Surveys International College Student Project reported that of ~14,000 full time students across 9 countries, including the US, Mexico, Germany, Belgium, and South Africa, the 12-month prevalence for any mental health disorder was ~31% (Auerbach et al., 2018). Another reason we focus on students is because MDD is highly heterogeneous in terms of both etiology and the problems people experience (Fried, Flake, & Robinaugh, 2022; Kendler, 2012a; Zimmerman et al., 2015), and efforts to understand and predict depression onset are more likely to succeed in more homogeneous populations (Cai et al., 2015, 2020). Moreover, prediction projects such as WARN-D require large samples, which are feasible to recruit in student populations, and students have the skills to operate the smartphone and smartwatch applications required for remote participation.

Third, WARN-D should be feasible for implementation in real-world settings. This precludes repeated lab visits and costly, time-intensive measurement such as brain scans and other biomarkers, which also do not appear to robustly predict depression onset (Border et al., 2019; Kennis et al., 2020; Winter et al., 2022). We instead focus on types of data that can readily be collected in the daily lives of students, including self-report surveys collected via smartphones, smartwatch data, and registry data. We will investigate the feasibility of our data collection protocol by querying participants about perceived burden of and barriers to participation.

Fourth, we aim to build a generic infrastructure that can be transferred and applied to many other disorders and target populations. If successful, WARN-D may spawn a host of follow-up projects that use the same infrastructure to provide personalized prediction of e.g., PTSD in military personnel, burnout in at-risk teachers, or manic episodes in recovered patients with bipolar disorders at risk for relapse. This promises to answer important scientific questions about personalized prediction across a range of mental disorders, such as which risk factors are transdiagnostic, which risk factors are disorder-specific, and which risk factors are specific to people with certain (e.g., demographic) features. This, in turn, relates to the identification of potentially novel mechanisms of change to inform future prevention programs (Nock, 2007).

Fifth, our study is guided by open scholarship principles. We are excited to make our design, measures, code, and data available to the research community. Information on design and measures are available in the accompanying Supplementary Materials. All empirical papers will be accompanied by open code; and we are currently developing a data sharing protocol with all relevant stakeholders which will be ready in 2025/2026 by the time data collection is finished. See our WARN-D project hub for all future updates and publications.

The final principle driving our design, methods, and measures is to conceptualize depression consistent with what we have learned about the complexities of the construct in the last decades (Fried, Flake, & Robinaugh, 2022). The next section is dedicated to this challenge.

## Conceptualization of Depression

Depression is a complex construct, and any study aiming to understand and predict MDD onset must grapple with these complexities. Challenges include (1) the heterogeneity of MDD in terms of etiology and symptoms; (2) depression severity as a continuum; (3) inter-individual differences of people diagnosed with MDD; (4) and the dynamic nature of MDD. We discuss these one by one below, and address how we aim to tackle them in WARN-D.

### Heterogeneity of Risk Factors and Symptoms

MDD is highly multifactorial, with many identified risk factors, all of which explain comparably little variance in isolation (Kendler, 2012a). Depression is also highly multi-faceted: common rating scales for depression encompass over 50 separate symptoms (Fried, Flake, & Robinaugh, 2022), and there is increasing evidence that symptoms are not interchangeable (Fried & Nesse, 2015b). For example, specific individual symptoms feature differential relations to constructs including impairment (Fried & Nesse, 2014; Tweed, 1993), biological markers (Frank et al., 2021; Fried et al., 2020; Hilland et al., 2020; Nagel et al., 2018; Van Eeden et al., 2020), life events (Fried et al., 2015; Keller et al., 2007; Keller & Nesse, 2005), and treatments (Boschloo, Bekhuis, et al., 2019; Boschloo, Cuijpers, et al., 2019; Snippe et al., 2021). Further, there is evidence that depression is not unidimensional, i.e., cannot be adequately described as *one* process (Fried et al., 2016). Together, this calls into question the practice of modeling depression as a single variable or process, and its etiology as driven by a small number of factors. Instead, it suggests the study of a broad set of biological, psychological, and social risk factors, protective factors, as well as problems or symptoms participants experience nested under the umbrella of the depressive phenotype (Engel, 1977).

### Depression Severity as a Continuum

Case-control studies are commonplace in depression research, where 2 groups (healthy vs depressed) are compared. This is widely recognized as a fundamental barrier to insights (Fried, Flake, & Robinaugh, 2022; Hitchcock et al., 2022), and categorical conceptualizations ignore subclinical cases who have increased levels of functional impairment, socioeconomic burden, service use, suicide attempts, and worse prognosis (Cuijpers & Smit, 2004; Gotlib et al., 1995; Hetrick et al., 2008; Judd et al., 1997). Dimensional perspectives in which subclinical cases are not subsumed into the category of healthy individuals offer ways forward that conceptualize depression as a continuum between healthy and sick, and align with evidence that depression behaves as a continuum at the between-subjects level (Conway et al., 2019; Haslam, 2003; Haslam et al., 2012), rather than a category or taxon.

### Inter-Individual Differences Within MDD

People diagnosed with MDD often differ from each other in fundamental ways regarding symptoms and etiology, and subsuming them into one group can obfuscate pronounced inter-individual differences (Fried, Flake, & Robinaugh, 2022; Fried & Nesse, 2015a; Kendler, 2012b; Zimmerman et al., 2015). Two patients can have the same DSM-5 diagnosis of MDD without sharing a single symptom, and knowing that a person is diagnosed with MDD tells us little about the actual problems they face in daily life (McWilliams, 2021; Parker, 2005). Longitudinal data combined with statistical approaches that can leverage such data efficiently (e.g., network models, machine learning models) allow researchers to disentangle group-level processes (i.e., the nomothetic) from personalized processes (i.e., the idiographic) in order to find out to which degree processes are shared across people (Fisher et al., 2018).

### Depression as a Dynamic Phenotype

This leads to the next challenge: the dynamic nature of depression (Hetrick et al., 2008; Judd et al., 1998; Wichers, 2014). There is sparse data on the nature of transitions into depression in the first place: are they largely categorical (i.e., a catastrophic transition), continuous (i.e., a process that unfolds slowly over weeks), or are there considerable inter-individual differences in how people transition into depression? Further, comparably little empirical work has been conducted on the depressive prodrome: what are the prominent features that could serve as early warning signals (EWS) for upcoming transitions into depression? Studies have identified a host of prodromal signs such as anxiety, sleep disturbances, worthlessness, sad mood, and concentration problems (Fava & Tossani, 2007; Iacoviello et al., 2010; Murphy et al., 2002), but results are inconsistent across studies, and prospective studies in large samples, including a long period of daily assessments, do not exist. One of the most comprehensive studies on the topic collected data every 6 weeks (Iacoviello et al., 2010), but cannot provide insights into daily fluctuations of problems. Such dynamic challenges require dynamic data, including daily reports of experiences, affect states, problems, and contextual variables whose fluctuations may shed light on upcoming transitions (Kuppens, 2015; van de Leemput et al., 2014; Wichers et al., 2016).

### WARN-D Embraces the Complexity of Depression

In sum, depression is a complex, dynamic, heterogeneous phenotype. To embrace this complexity, WARN-D is guided by the rationale of depression as emerging from a system of biopsychosocial elements (Fried, 2022), which we term the *human mood system.* We conceptualize this system broadly, including time-invariant (or very slow-moving) features such as personality; time-varying features such as a person’s thoughts, feelings, and behaviors; as well as the context in which experiences are made; a detailed list of all assessed features is provided later. Understanding this human mood system and its development requires the study of a broad set of system elements as well as their interrelations (Borsboom, 2017; Olthof et al., 2023), which is why we use smartphones and smartwatches to gather dynamic data. Conceptualizing complex processes as multivariate, multicausal systems has resulted in many breakthroughs in disciplines such as ecology, meteorology, medicine, public health, and social dynamics (Barabási, 2012; Castellano et al., 2009; Luke & Stamatakis, 2012; Olde Rikkert et al., 2016; Quax et al., 2018). In clinical psychology and psychiatry, recent studies have demonstrated the potential utility of a systems approach for understanding mental health problems like depression (Hayes & Andrews, 2020; Lutz et al., 2018; Olthof et al., 2023; Robinaugh et al., 2020; Wichers, 2014). Of particular interest are EWS that have been uncovered in many different areas of research, showing that systems close to transitions into alternative states (e.g., from healthy states to disordered states) show particular behavior that can be leveraged to forecast upcoming transitions (Olthof et al., 2020; van de Leemput et al., 2014; Wichers et al., 2016).

WARN-D hopes to identify such markers in the human mood system to predict individuals at risk for imminent system shifts into depression. In our communication with participants, we use weather forecasting and thunderstorms as a metaphor for this: thunderstorms are not best predicted by increases in thunderstorms, and in the same way, monitoring symptoms over time may not be the best way to predict depression onset. Instead, thunderstorms are best predicted by monitoring features of the weather system, along with the dynamic relations among these features. Together, these can provide evidence of upcoming changes in the system. The main difference between forecasting thunderstorms and depression is that in the former case, if we successfully anticipate an upcoming storm, all we can do is to accept the incoming storm, seek shelter, and try to clean up afterwards. For depression, successful prediction may allow us to prevent depression before it occurs in the first place.

## WARN-D Design, Procedure, and Measurement

### Design

**Figure 1:**
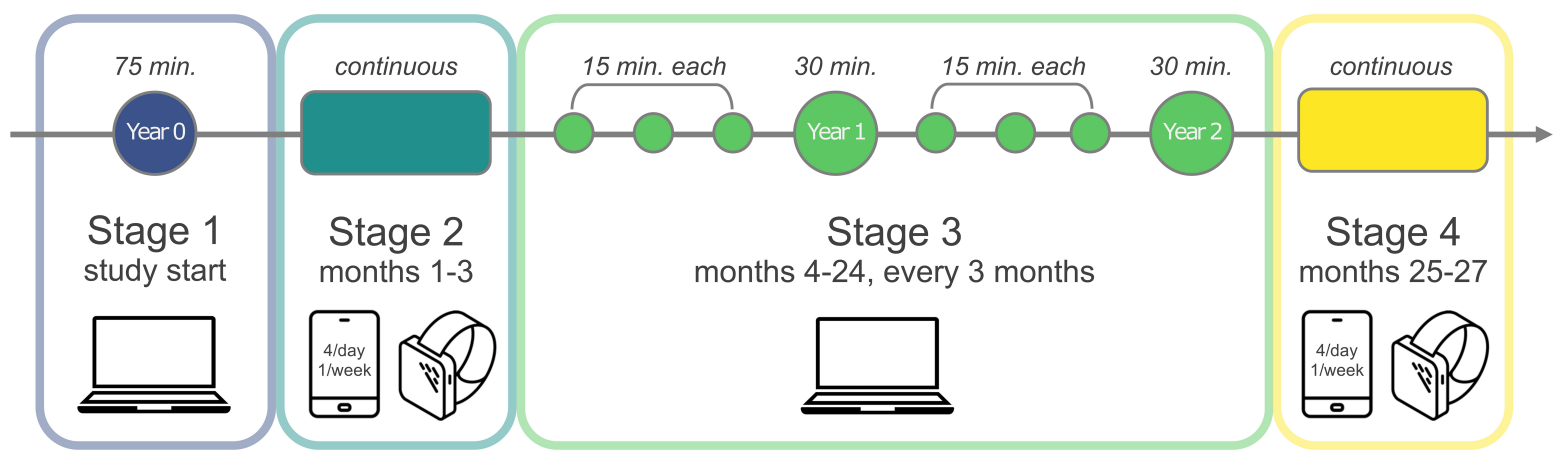
Overview of Design and Procedure of the WARN-D Study *Note.* The study takes place in 4 cohorts with a target n = 500 per cohort, and each cohort runs for 2 years through Stages 1, 2, and 3. Starting times for cohorts are November 2021, May 2022, November 2022, and May 2023. For Stage 4, the repetition of Stage 2, we will re-invite all participants from cohorts 1 and 2. Attribution of images: laptop, phone, and smartwatch by Mello, Rabi'ah Al Adawiyyah, and Smashicons, respectively (Noun Project, CC BY 3.0).

We plan to follow 2,000 students from vocational schools, technical universities, and universities in the Netherlands for ~2 years, using a multicohort design with 4 cohorts of 500 students each. The 4 cohorts start in November 2021, May 2022, November 2022, and May 2023, respectively. The timeline of the project is visualized in Figure 1 and includes 4 stages.

After participants meet inclusion criteria based on a brief online screener, Stage 1 consists of a 75-minute online survey with the goal to assess risk factors for depression broadly. Stage 2 collects daily smartwatch and smartphone data, obtaining detailed insights into students’ lives. Stage 3 consists of 8 online surveys, every 3 months, to determine if changes in mental health have occurred, and to assess risk and resilience factors.

The study officially terminates after Stage 3, at which point we plan to re-invite all participants from cohorts 1 and 2 for Stage 4, which is a repetition of Stage 2, i.e., another 3 months of daily monitoring. Study design, procedure, inclusion and exclusion criteria, and measurement are described in more detail in the Supplementary Materials.

### Procedure

The WARN-D study is funded by the European Research Council under the European Union’s Horizon 2020 research and innovation program (No. 949059). The data collection was approved by the Leiden University Research Ethics Committee Leiden (2021-09-06-E.I.Fried-V2-3406). The study was exempted from having to obtain ethics approval under the Medical Research Involving Human Subjects Act.

Although data collection is finished for some cohorts, it is still running for others; therefore, we will use present tense in the remainder of the procedure section. We advertise the study both online and offline, and partnered with several initiatives (e.g., Caring Universities) and educational institutions (e.g., MBO Rijnland) to reach students. Participants interested in participating receive a link to an online survey. Upon signing up, they can choose their preferred language (Dutch or English), and then read and sign the informed consent materials. After a screener on inclusion and exclusion criteria described below, participants are invited to Stage 1 of the study; completing Stage 1 is mandatory to be invited to Stage 2.

We pay participants up to 90€ for completing all surveys in Stage 1 (7.50€), Stage 2 (45€), and Stage 3 (37.50€; 7.50€ for the 30-minute surveys at 12 and 24 months, and 3.75€ for the 15-minute surveys at 3, 6, 9, 15, 18, and 21 months). Participation in Stage 4 yields up to 45€. Participants who complete the 1-year and 2-year follow-up surveys in Stage 3 can participate in 500€ lotteries for each survey. Further, participants completing Stage 2 receive a personalized report of the self-report data collected via smartphones, based on our experiences in a recent study that this is of great interest to many participants (Fried, Papanikolaou, & Epskamp, 2022). 

### Inclusion and Exclusion Criteria

Participants qualify for the study if they meet the following criteria: ≥18 years old; fluent in reading Dutch or English; studying at a Dutch educational institution pursuing an MBO (vocational school), HBO (higher vocational school), or WO (university) degree (no PhD students); currently living in the Netherlands, Germany, or Belgium (this is to ensure that smartwatches can be shipped in time); having a European bank account (for reimbursement purposes); and having a smartphone that runs on Android or iOS so that the apps required for Stage 2 work without problems.

Participants are excluded if they meet any of the following 6 criteria. First, at least moderate levels of current depression, operationalized via a score of ≥2 on the 2-item Patient Health Questionnaire (PHQ-2; Kroenke et al., 2003) and then a score of ≥14 on the 9-item Patient Health Questionnaire (PHQ-9; Kroenke & Spitzer, 2013). Second, current mania, operationalized via the corresponding items on the American Psychiatric Association’s (APA) “DSM-5 Self-Rated Level 1 Cross-Cutting Symptom Measure—Adult” (Narrow et al., 2013), from here on referred to as the Level 1 screener, followed by APA’s recommended Level 2 screener, the Altman Self-Rating Mania Scale (Altman et al., 1997); participants are excluded if they meet thresholds on both Level 1 (≥2 on either of the 2 items) and Level 2 (sum score ≥6) screeners. Third, current thought disorders, operationalized via the Level 1 screener (sum score ≥1). Fourth, substance use disorder via the Alcohol, Smoking and Substance Involvement Screening Test (ASSIST v3.0), using the cutoff of ≥27 for each substance (WHO ASSIST Working Group, 2010). Fourth, we exclude participants reporting that they are currently in treatment or waiting for treatment for the mental health problems described above. Fifth, we exclude students with at least moderate current suicidal ideation, operationalized via a score of 2 on item 4 of the Beck Scale for Suicide Ideation (BSS; Beck et al., 1979), which has shown excellent psychometric properties to screen for suicidal ideation, including in a Dutch sample (De Beurs et al., 2014). Finally, we exclude participants who indicate that they would find seeing an estimate of daily calories burned very stressful, given that the smartwatches worn in Stage 2 provide such an estimate. 

### Measurement

There are many tools to measure constructs in clinical psychology and psychiatry. For our baseline and follow-up assessments, we based our selection of measures on 5 guiding principles:

1. Scales should assess constructs relevant to understanding the human mood system and predict changes of the system (e.g., protective and risk factors).
2. Scales should be free to use and in the public domain.
3. Scales should have adequate psychometric properties.
4. Scales should be validated in both English and Dutch.
5. Scales should be short without sacrificing content validity.

Some of the measures had to be created, translated, or adapted. Guiding principles for measure adaptation were:

1. Adapt as little as possible.
2. Minimize burden for participants. We did so by streamlining time periods (e.g., we adapted the Perceived Stress Scale (Cohen & Williamson, 1988) from the last 4 weeks to the last 2 weeks, so it is aligned with all our other measures that capture 2 weeks); by shortening scales to remove items not of interest to our research; and by shortening repetitive instructions (e.g., many scales instruct participants to “read these items carefully”).
3. Adapt measures to ensure they are adequate for most participants in a student sample in the Netherlands. Three examples are: we changed the unit “stone” to “kilogram” in the SCOFF scale (Morgan et al., 1999); we removed the item “combat or exposure to a war-zone” from the Life Events Checklist for DSM-5 (Gray et al., 2004); and we replaced the examples “gardening”, “collecting”, and “sewing” with “playing computer games” in the leisure domain of the Work and Social Adjustment Scale (Mundt et al., 2002).

Because our selection of constructs may miss important aspects of participants’ lives, every stage affords participants the opportunity to indicate further relevant information in open text fields. Overall, we have made all questionnaires and codebooks for all measures available in the Supplementary Materials. 

#### Stage 1: Baseline

Stage 1 consists of a 75-minute Qualtrics survey to collect research data. Table 1 contains an overview of our measurement battery, resulting from a detailed literature review and several expert meetings, followed by a short Delphi study with 12 clinicians and researchers from clinical psychology and psychiatry.

**Table 1:** Stage 1 (Baseline) Measurements in the WARN-D Study

Category	Examples
Demographics	Age, nationality, population group
Physical appearance	Height, weight and satisfaction therewith, satisfaction physical appearance
Sex and gender	Biological sex, gender identity and struggles, sexual orientation and struggles
Internationality	Time spent in the Netherlands, integration into Dutch Society, international student status
SES and finances	Subjective socioeconomic status, current work, income sources, income vs spending, satisfaction work and finances, parents’ and own highest education
Education	Current studies and satisfaction, academic standing and satisfaction
Living situation	Children, household composition, satisfaction living situation
Religion	Religious affiliation, connection to church, place of worship
Physical health	Global physical health and impairment rating last 2 weeks and last year, chronic health issues, pain, medication
Menstruation-related questions	Detailed menstruation information, pregnancy / breastfeeding, contraception
COVID-19	Impact of pandemic on mental health, prior COVID-19 diagnoses, COVID-19 symptom severity, long COVID-19 symptoms
Sleep habits	Chronotype, sleep schedule, sleep problems like nightmares, worry about sleep, impairment, satisfaction
Mental health	Family history, global mental health and impairment rating last 2 weeks and last year, lifetime emotional problems, current / prior problems and diagnoses, recent changes in mental health, current need for treatment, current / prior treatment, current and lifetime depression, current seasonal affective disorder / (hypo)mania / generalized anxiety disorder / social anxiety disorder / obsessive-compulsive disorder / eating disorder / borderline personality disorder, current and past suicidal ideation, prior suicide attempts, non-suicidal self-injury
Substance use	Current, past, and lifetime substance use problems
Wellbeing and stressors	Hedonic and eudaemonic wellbeing, general life satisfaction, current stress and stress domains, childhood and lifetime adversity, discrimination, bullying, feelings of safety, negative and positive life events
Social	Social network online / offline, social media use, positive / negative interpersonal experiences, satisfaction relationship with friends / family, relationship status and satisfaction, satisfaction sex life, satisfaction independence from parents, loneliness
Leisure and activity	Physical activity, sedentary behavior, time spent outside, leisure activities and satisfaction
Traits and tendencies	Attachment style, negative affect, big five personality traits, repetitive negative thinking, intolerance to uncertainty, pessimism, behavioral and cognitive emotional regulation strategies, affective lability, anger/irritability, perfectionism, workaholism, dependency/separation anxiety/insecurity, procrastination
Resilience	Perceived stress recovery, self-efficacy, self-esteem, locus of control
Meta	Motivation to participate, survey difficulty, attention paid while answering, feedback on survey
Registry data	Air pollution, educational facilities, green spaces, income, urbanization, traffic noise, poverty, value of houses

*Note.* For a full list of variables, phrasing, response options, translations, and bibliography of measurement instruments, see codebook in the Supplementary Materials.

In addition to this survey, we ask participants for permission to link their postal code to Dutch registry data containing neighborhood information such as air pollution, green spaces, and traffic noise (see Table 1); such data may be helpful as indicators for socioeconomic status, which in turn has been shown to be related to depression (Platania, 2023). More information about registry data is available at gecco.nl; permission to link postal code to registry data is not necessary for participation in WARN-D.

#### Stage 2: Daily Monitoring

Stage 2 aims to provide a detailed mapping of the biopsychosocial components of the human mood system. This includes the temporal dynamics of important variables like depression and anxiety symptoms, affect states, stress, functional impairment, activity, sleep, as well as contextual variables.

To assess these data, we use ecological momentary assessment (EMA) to follow participants in their daily lives via smartphones for 85 days (Bos et al., 2019; Larson & Csikszentmihalyi, 1983; Myin-Germeys & Kuppens, 2021). Specifically, we use the Ethica app to query people 4 times a day, between around 10 am and 9:30pm at intervals of around 225 minutes with a normally distributed 30-minute jitter for each survey; each survey expires after 20 minutes. All 4 surveys contain the same block of 18 questions and take about 1-2 minutes to complete. The morning survey contains 3 additional questions about the last night and outlook for the day, and the evening survey contains 18 additional questions about the day as a whole. In addition, we query people every Sunday at noon for a 46-item survey that takes around 5-7 minutes to complete, expiring after 10 hours. Table 2 summarizes EMA measurement design and content. An example item is “How sad are you right now”, which we query using a 7-point Likert scale from 1 (not at all) to 7 (very much). Our measures are based on the literature, our prior work, currently ongoing projects, and discussions with EMA experts. The number of prompts and items per prompt were chosen based on discussions with numerous experts as well as our own experience regarding the compliance rates of EMA data in student populations, with the overarching goal to obtain insightful momentary data whilst ensuring that the EMA protocol is feasible for students; for that reason, we also assess if participants experience the monitoring as burdensome.

**Table 2:** Stage 2 Measurements in the WARN-D Study

Category	Examples
Mental health	Stress and stress domains, mental health and interference with daily activities, depression and anxiety symptoms, bad dreams, non-suicidal self-injury
Positive and negative affect	Happy/cheerful, motivated, relaxed, stressed, sad, nervous/anxious, overwhelmed, annoyed/irritated
Satisfaction and wellbeing	Ability to concentrate, feeling productive, general satisfaction
Physical experiences	Physical health and interference with daily activities, pain/discomfort, sleep, substance use, menstruation, sleep and tiredness
Experiences	Best and worst experiences of the day and the week, category of experiences such as finances, education, and love life
Social experiences	Feeling connected to others, being able to rely on others for support, current social offline/online contact, social media use
Context	Current activity and enjoyment of activity, current location
Coping and Appraisal	Being able to handle daily and weekly challenges, emotion regulation
Meta	Enjoying study participation, reasons for missing surveys
Garmin smartwatch	Heart rate (constant, daily resting), blood oxygen saturation monitor, energy monitor, stress, body battery, sleep, step counter

*Note.* For a full list of variables, phrasing, response options, and translations, see codebook in the Supplementary Materials.

We also collect digital phenotype data via the Garmin smartwatch Vivosmart 4, including sleep phases and duration, activity, heart rate, and stress.

#### Stage 3: Follow-Up Surveys

Stage 3 consists of 8 follow-up surveys. Two of these (the yearly ones) last ~30 minutes, the others ~15 minutes; see Table 3 for an overview of the assessed constructs.

**Table 3:** Stage 3 (Follow-Up) Measurements in the WARN-D Study

Category	Variables
Physical appearance	Height, weight and satisfaction therewith, satisfaction physical appearance
Sex and gender	Struggles with gender identify / sexual orientation
Internationality	Integration into Dutch Society
SES and finances	Satisfaction work and finances, highest education
Education	Current studies and satisfaction
Living situation	Children, satisfaction living situation
Physical health	Global physical health and impairment rating last 2 weeks and last 3 months, medication
Menstruation-related questions	Pregnancy/breastfeeding, contraception
COVID-19	Impact of pandemic on mental health, prior COVID-19 diagnoses, COVID-19 symptom severity, long COVID-19 symptoms
Sleep habits	Sleep, nightmares, satisfaction
Mental health	Global mental health and impairment rating last 2 weeks and last 3 months, current emotional problems and diagnoses, recent changes in mental health, current need for treatment, current treatment, current depression, current generalized anxiety disorder, current/prior suicidal ideation, suicide attempts, non-suicidal self-injury
Substance use	Current substance use habits
Wellbeing and stressors	Hedonic and eudaemonic wellbeing, current stress and stress domains, negative and positive life events
Social	Social network online / offline, social media use, positive / negative interpersonal experiences, satisfaction relationship with friends / family, relationship status & satisfaction, satisfaction sex life, satisfaction independence from parents, loneliness
Leisure and activity	Physical activity, satisfaction leisure activities
Traits and tendencies	Neuroticism, behavioral and cognitive emotional regulation strategies, affective lability, perfectionism, dependency/separation anxiety/insecurity, procrastination
Resilience	Perceived stress recovery, perceived recent resilience, forecast resilience
Meta	Motivation to continue participation, feedback on survey

*Note.* For a full list of variables, phrasing, response options, translations, and bibliography of measurement instruments, see codebook in the Supplementary Materials.

#### Stage 4: Repetition of Stage 2

Stage 4 is a repetition of Stage 2. We aim to recruit ~500 participants from Cohorts 1 and 2 who previously completed Stage 2 to obtain insight into the temporal stability of the human mood system.

## Strengths and Challenges

We hope to achieve our ambitious goal of building a personalized early warning system for depression by embracing the complexity of the human mood system (Fried, 2022; Olthof et al., 2023). A multi-disciplinary approach integrating advances from systems theory, multi-modal measurement, and statistical models will be crucial to achieve this aim. The project also faces several challenges, and many open questions remain.

First, there is a large literature on EWS in other disciplines such as ecology (Dakos et al., 2012; Scheffer et al., 2012), and the psychological literature is growing rapidly (e.g., Adler et al., 2020; Cabrieto et al., 2019; Olthof et al., 2020). Which EWS may be predictive of depression remains to be seen, and we will focus on both data-driven and theory-driven EWS by leveraging all collected data and using machine-learning models to analyze what particular features are predictive of an upcoming transition, but also by testing various EWS proposed in the literature. One is critical slowing down, a feature that has been shown to predict transitions in systems such as lakes before they turn from clean to turbid states, non-linear physical systems such as Earth’s climate, as well as the stock market (Olde Rikkert et al., 2016; Quax et al., 2018; Scheffer et al., 2012, 2018). Slowing down is a marker that a system becomes more vulnerable for an upcoming transition, because vulnerable systems take longer to recover from perturbations, which goes together with changes in parameters of systems that can be observed. Another EWS is higher connectivity, defined as more and stronger relations among components in a system, which could confer vulnerability for future depression. This is because in a more strongly connected causal system of problems (e.g., sleep problems, sad mood, concentration problems, fatigue), activating one problem may lead to a cascade that activates others (Cramer et al., 2016; Schweren et al., 2018; van Borkulo et al., 2015; van de Leemput et al., 2014).

A second challenge is that WARN-D is focused on forecasting depression, a complex and fuzzy phenotype for which many defensible operationalizations exist. For this reason, we will predict several outcome variables, rather than restricting ourselves to one arbitrary operationalization. Outcomes include: stress, anxiety and depression severity, as well as probable MDD diagnosis; wellbeing and impairment of functioning; changes in any of these constructs over time (as observed by longitudinal data), as well as perceived changes in these outcomes over time as retrospectively reported by participants. Further outcomes include self-report information participants provide on diagnoses by health care professionals, as well as starting psychological or pharmacological treatments for MDD or related conditions. A robust predictor is one that predicts a larger number of these operationalizations of significant mental health changes.

Third, attrition rates are a concern in EMA studies and prospective studies. To mitigate attrition, we incentivize participants in various ways: we pay them per completed survey (up to 90€ in total); organize 500€ lotteries per cohort for completing the 1-year and 2-year surveys, respectively; provide participants with Garmin VivoSmart 4 smartwatches they can use freely in Stage 2; and offer participants a personalized data report of their EMA data after completing Stage 2. We also continuously ask participants about their experience with WARN-D to learn about participation barriers with the goal of minimizing attrition rates in future cohorts.

Finally, WARN-D is an observational study, since our primary goal is prediction of onset—interventions carried out by WARN-D itself would stand in the way of accurate forecasting. However, one could argue that especially Stage 2 (tracking people via smartphones and smartwatches) may itself be an intervention. Fortunately, we hope that such effects are held constant across time. That is, after we have developed the WARN-D app in a few years to predict onset, people using it will go through a very similar program as described here, tracking themselves via smartphones and smartwatches to collect data to enable prediction of future onset. The app will likely also support a functionality where users can view the data they provide, similar to the personalized data reports. In that sense, our observational validation cohort for WARN-D, and the people using the app in the future, will receive similar self-tracking ‘interventions’, holding potential intervention effects constant in our prediction and validation samples.

## Supplementary Materials

Supplementary Materials are available online (Fried, Proppert, & Rieble, 2023a), which contain further information regarding:

1. Consent sheets and general information sheets for participants, inclusion and exclusion screener, codebook for the screener
2. Design and procedures for all stages
3. All questionnaires and codebooks in Dutch and English for all stages
4. Stage 1: data journey for participant data, mental health information package for participants, information on the delphi study, information on the registry data
5. Stage 2: participant instruction materials and video for setting up smartphones and watches
6. Procedures for the personalized data reports and an example report

FriedE. I.
ProppertR. K. K.
RiebleC. L.
 (2023a). Supplementary materials to "Building an early warning system for depression: Rationale, objectives, and methods of the WARN-D study"
[Additional information]. PsychOpen. 10.17605/OSF.IO/2JD9H
PMC1086364038356901

FriedE. I.
ProppertR. K. K.
RiebleC. L.
 (2023b). Supplementary materials to "Building an early warning system for depression: Rationale, objectives, and methods of the WARN-D study"
[Project hub]. PsychOpen. 10.17605/OSF.IO/FRQDV
PMC1086364038356901

## Data Availability

We are excited to make our design, measures, code, and data available to the research community. Information on design and measures are available in the accompanying Supplementary Materials (Fried, Proppert, & Rieble, 2023a). All empirical papers will be accompanied by open code; we are currently developing a data sharing protocol with all relevant stakeholders which will be ready in 2025/2026 by the time data collection is finished. See our WARN-D project hub (Fried, Proppert, & Rieble, 2023b) for all future updates and publications, and, eventually, data.
